# Supplementary material for: Splice-Junction-Based Mapping of Alternative Isoforms in the Human Proteome
Source: Cell Rep. Author manuscript; Available in PMC 2020 Jan 15. (PMC6961840; doi:10.1016/j.celrep.2019.11.026)
Supplement: 2 [file NIHMS1546469-supplement-2.zip › DF1/PXD010154/Testis/Testis_47_CPT1B_RPHQEVPDQPFQFTVTPDGVDFR.pdf]

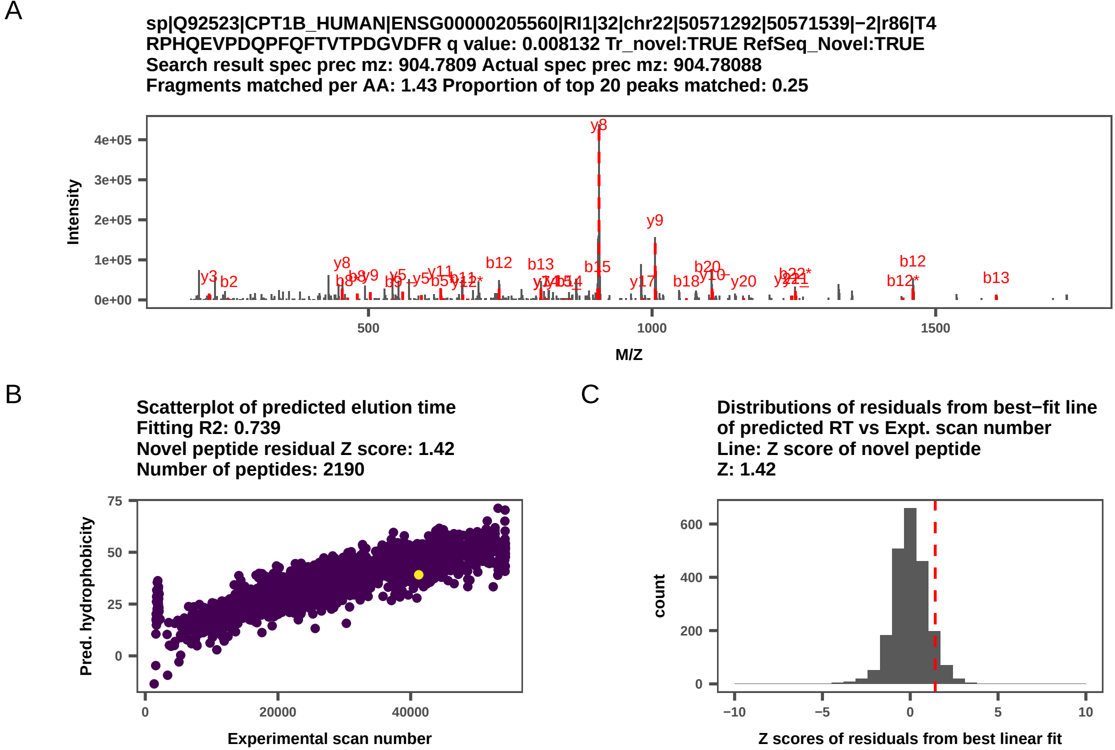
A
sp|Q92523|CPT1B_HUMAN|ENSG00000205560|RI1|32|chr22|50571292|50571539|−2|r86|T4
RPHQEVPDQPFQFTVTPDGVDFR q value: 0.008132 Tr_novel:TRUE RefSeq_Novel:TRUE
Search result spec prec mz: 904.7809 Actual spec prec mz: 904.78088
Fragments matched per AA: 1.43 Proportion of top 20 peaks matched: 0.25
Intensity
4e+05
3e+05
2e+05
1e+05
0e+00
y8
y9
y3
b2
b12
b13
b15
y17
b18
y20
b12
b12*
b13
500
1000
1500
M/Z
B
Scatterplot of predicted elution time
Fitting R2: 0.739
Novel peptide residual Z score: 1.42
Number of peptides: 2190
Pred. hydrophobicity
75
50
25
0
0
20000
40000
Experimental scan number
C
Distributions of residuals from best−fit line
of predicted RT vs Expt. scan number
Line: Z score of novel peptide
Z: 1.42
count
600
400
200
0
−10
−5
0
5
10
Z scores of residuals from best linear fit
